# Editorial: Advances in nanomedicine and drug delivery approaches in inflammatory-diseased conditions

**DOI:** 10.3389/fbioe.2026.1962389

**Published:** 2026-08-18

**Authors:** Gesmi Milcovich, Maurizio Pesce, Filipe E. Antunes

**Affiliations:** 1 Department of Life Sciences, University of Modena and Reggio Emilia, Modena, Italy; 2 Department of Medicine, LUM University, Casamassima (BA), Italy; 3 Unità di Ingegneria Tissutale Cardiovascolare, Centro Cardiologico Monzino, IRCCS, Milan, Italy; 4 Department of Aerospace and Mechanical Engineering, Turin School of Engineering, Turin, Italy; 5 Department of Chemistry, Coimbra Chemistry Centre, University of Coimbra, Coimbra, Portugal

**Keywords:** biological bases for inflammation, drug delivery, high-impact diseases, inflammatory mechanisms, nanomedicine, stimuli-responsiveness, systems biological approaches on biomedicine

Nowadays, most high-impact diseases, such as cancer, vascular, neurodegenerative diseases and high pain conditions displayed a shared feature: inflammation, oxidative damages and related mechanisms. Hence, in order to target the key hallmarks of such conditions, a set of innovative approaches is required, in order to fully understand the pathological signals and address them properly. In the context of such a scenario, investigating the molecular mechanisms and biological cues associated with different diseased conditions is of utmost importance. Indeed, the identification of the inflammatory cascade throughout different pathological models is the first milestone that can lead to understand and propose finely tunable and targeted drug delivery and treatments.

The aim of the present Research Topic was focused on the Research Topic of both experimental work and review articles related to inflammatory mechanisms and their direct correlation with high-impact diseases, such as cancer, vascular issues/atherosclerosis, and neurodegenerative conditions.

In a valuable contribution from Isma Liza Mohd Isa group (Kamali et al.) an elegant perspective is presented, in order to address intervertebral disc regeneration, which is a major cause of lower back pain condition and inflammation. Indeed, they proposed the application of engineered extracellular matrix-based hydrogels, considering the intervertebral disc regeneration is linked to a dysregulation of extracellular matrix (ECM) and inflammation. Therefore, by mimicking the ECM, newly developed hydrogels proposed a promising option for the development of long-lasting, effective therapeutic solutions. Hence, these hydrogels can deal with the main causes of disc degeneration and allow the perspective of new treatment for individuals living with chronic lower back pain condition.

Inflammation and related mechanisms have been thoroughly examined by He et al., on their interesting review, dealing with inflammatory bowel disease as a chronic, non-specific intestinal inflammatory condition. They mainly point out the importance of nanotechnology and nanomaterials approaches in order to address the inflammation. In detail, aromatic rings were zoomed in for their ability to provide for drug stability, improve drug solubility and targeting, while exerting anti-inflammatory, antioxidant, and immunomodulatory features. Such approaches were proposed as a leader innovation for inflammatory bowel disease treatment.

On another key contribution from Li et al., a thorough examination of a high impact health issue, like early diagnosis of acute rejection in heart transplantation, was provided. Moreover, a focus on the upfront development of nanomaterials in such context was discussed, including all possible challenges and future directions for the clinical translation of nanomaterials in heart transplantation. Nanomaterials were pointed out thanks to their innovation and potential as drug delivery systems. Furthermore, they can serve as a platform to add specific ligands, which can enhance drug delivery, boost therapeutic effects, while decreasing adverse reactions events. Moreover, nanomaterials are presented as theranostic agents, able to behave as finely targeted molecular probes to support several imaging techniques, in order to assist the monitoring process of e.g., the infiltration of immune cells (such as T cells and macrophages) in severe cardiac issues.

An experimental contribution from Gaia Spinetti group (Campanile et al.) interestingly revealed the pivotal role of inflammatory processes linked to cardiovascular diseases and their mechanisms understanding need, in order to properly address this kind of high impact conditions. Specifically, they focused their study on age-associated changes in the bone marrow, as a key signal of cardiovascular issues. Indeed, an impaired mobilization of bone marrow-derived cardioprotective CD34^+^ hematopoietic stem/progenitor cells proved to be a cause for a lower frequency in the circulation and poorer cardiovascular outcomes. Their findings and approach aimed to create a milestone for translational biomedical research, while proposing future test of the presented model, in the context of human-relevant drug screenings.

Finally, an additional article from Maan et al. further focused on the immune responses and their pathological and molecular mechanisms, directly connected to the inflammation cascade. Disruption of the skin barrier, in this case, whether due to surgery, traumatic events, burns or chronic metabolic disease leads to a healing cascade. This process is directly connected to inflammatory signaling, extracellular matrix remodeling, angiogenesis and coordinated cellular responses. In acute wounds, it usually progresses with a set of orderly phases of repair. On the other hand, in chronic wounds (e.g., diabetic foot ulcers, venous ulcers and pressure injuries) the remodelling and immune responses are impaired by strong inflammation cascades. The inflammation results in protease imbalance, senescent cell accumulation, impaired vascularization and colonization of microbial biofilms, hence conducting to the development of co-morbidity and multiple healthcare burden.

Overall, all publications included within this Research Topic provided for an in-depth overview of the recent advances in terms of biochemical, mechanobiological, and biological signals to the cells within inflamed tissue environment, as well as their nanotechnology solutions. The new approaches and applications of these outlooks indicate increasing efforts towards the reduction of high impact diseases, such as cancer, vascular diseases, and neurodegenerative conditions, and push forward new therapeutic options.

